# Confocal imaging dataset to assess endothelial cell orientation during extreme glucose conditions

**DOI:** 10.1038/s41597-022-01130-x

**Published:** 2022-01-27

**Authors:** Ana María Porras Hernández, Laurent Barbe, Hannah Pohlit, Maria Tenje, Maria Antfolk

**Affiliations:** 1 Department of Materials Science and Engineering, Science for Life Laboratory, Uppsala University, Uppsala, Sweden; 2 Department of Biomedical Engineering, Lund University, Lund, Sweden; 3 Biotech Research and Innovation Centre, University of Copenhagen, Copenhagen, Denmark

**Keywords:** Type 2 diabetes, Actin

## Abstract

Confocal microscopy offers a mean to extract quantitative data on spatially confined subcellular structures. Here, we provide an imaging dataset of confocal z-stacks on endothelial cells spatially confined on lines with different widths, visualizing the nucleus, F-actin, and zonula occludens-1 (ZO-1), as well as the lines. This dataset also includes confocal images of spatially confined endothelial cells challenged with different glucose conditions. We have validated the image quality by established analytical means using the *MeasureImageQuality* module of the CellProfiler^TM^ software. We envision that this dataset could be used to extract data on both a population and a single cell level, as well as a learning set for the development of new image analysis tools.

## Abstract (summary)

**Table:** 

Measurement(s)	nucleus • F-actin • zonula occludens 1
Technology Type(s)	confocal microscopy
Factor Type(s)	line width • medium glucose concentration
Sample Characteristic - Organism	Mus musculus

Machine-accessible metadata file describing the reported data: 10.6084/m9.figshare.17129021

## Background & Summary

Cells are constantly exposed to various biomechanical signals throughout the body *e.g*., organ motion, fluid flow, or cell-extracellular matrix interactions. These external forces are converted into intracellular signals that affects the cell’s gene expression and subsequent cellular response^1^. Indeed, changes in cell shape is often associated with nucleus shape change as well, and have an effect on chromatin condensation and ultimately cell proliferation^2^. The effect of cell shape changes on the nucleus shape is mediated by cytoskeletal actin filaments, mainly regulated by the perinuclear actin cap, a dome-like actin structure that covers the top of the nucleus^3^. In this way, cell shape and orientation results in actin filament organization, which orients and shapes the nucleus.

Different disease states, among these diabetes, have been shown to affect the endothelial cell functions and thus might ultimately alter the barrier properties^4^. Indeed, altered glucose levels have been seen to contribute to this dysfunction, through *e.g*., disturbing the cell’s ability to align and regulate its shape^5^. However, remarkably little is currently known about what effects spatial confinement and altered glucose levels have on a cell’s shape, and orientation.

Microfabrication techniques have been extensively used in the electronics and microelectromechanical systems (MEMS) fields and their recent combination with the biomaterials field have paved the way for more advanced micropatterning opportunities of biomaterials^6^. It has been seen that the cells ability to adhere to a surface can be spatially controlled and confined to specific regions by micropatterning cell adhesion peptides or proteins to inert surfaces otherwise not allowing the cells to adhere^7^. In addition, it has been demonstrated that cell shape changes induced by spatial confinement to micropatterns alone is enough to alter gene expression, and thus that cellular shape determines cell functions^8,9^.

**Fig. 1:**
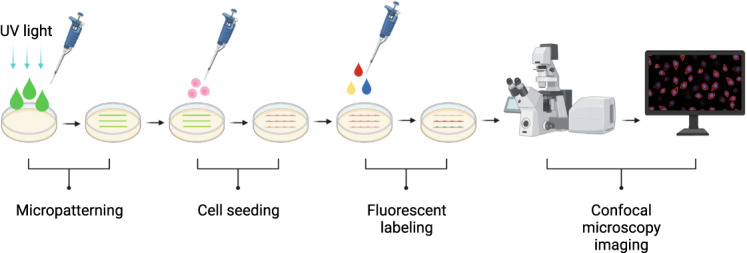
Method workflow. Hydrogels were formed from hyaluronic acid acrylamide precursor and patterned with RGD-peptide lines using UV light. Cells were then seeded on these gels and adhered exclusively to the lines. The cells were left in culture for 24 h before they were fixed and fluorescently labelled. Subsequently, the samples were visualized using confocal microscopy. Created with BioRender.com.

We recently developed a new method to micropattern hyaluronic acid acrylamide hydrogels with adhesion peptides and proteins^10^. To generate this dataset, we patterned RGD-peptides on the hyaluronic acid acrylamide hydrogels, in lines of different widths, varying between 10–100 µm (workflow illustrated in Fig. 1). We proceeded to seed brain endothelial microvascular cells on these lines and exposed the cells to both hypo- and hyperglycaemic conditions. We generated three datasets visualizing the cell membrane, cytoskeleton, and nucleus. Here, we also include a rigorous validation and detailed instructions as to how this experiment can be performed.

We envision that our confocal imaging dataset could be utilized by other researchers to extract data on both the population level and on a single cell level. The included confocal stacks allow for spatial localization within the cells. This dataset can also be used as a reference or comparison set for reapplying the same experimental conditions to another cell type *e.g*., another endothelial subtype, or applying similar but different experimental conditions to the same cell type *e.g*., other culture conditions or time points. Furthermore, this dataset can also be used as a training set for developing new image analysis tools or for building and evaluating new deep learning models. This dataset has been used in a previous publication, but we believe that this dataset has a high reuse value, and more extensive analysis could be performed using it^11^.

## Methods

### Synthesis of hyaluronic acid acrylamide derivative

Hyaluronic acid acrylamide was synthesized as has been previously reported elsewhere by us and others^10,12^. Sodium hyaluronate (Lifecore Biomedical) was functionalized with acrylamide groups by reacting the carboxylic acid group of HA to an amino group of *N*-(2-aminoethyl) acrylamide linker (abcr GmbH). The final product was freeze-dried and kept at −20 °C until use. The degree of modification was 14% as determined by ^1^H NMR.

### Forming and pattering hyaluronic acid acrylamide hydrogel

Patterned hydrogels were prepared as previously described by us^10,11^. Here, to form the hydrogels, Irgacure 2959 (Sigma-Aldrich) was dissolved in phosphate buffer to a final concentration of 0.4% (w/v). Next, hyaluronic acid acrylamide was dissolved in the Irgacure 2959 solution to a final concentration of 2.0% (w/v). This hydrogel precursor solution was placed in a Si/SU8 mould and exposed to UV light (4.6 J/cm^2^) to initiate cross-linking of the gel. A glass cover slip was used as a lid to the Si/SU8 mould to control the thickness of the resulting hydrogel. This resulted in hydrogels with 100 µm thickness.

The resulting hydrogel films were then covered by a solution of 0.5 mM 5FAM-GCGYRGDSPG peptide (Innovagen AB) and exposed to UV light through a photomask (1.9 J/cm^2^) to define the patterned areas. The photomask consisted of straight lines with varying widths (10, 25, 50, 75 and 100 µm) and constant spacing (100 µm). Control samples were prepared by linking the adhesion peptide to the whole gel surface. To do this the hyaluronic acid acrylamide hydrogel was covered with peptide solution 5-FAM-GCGYRGDSPG (green fluorescent) and exposed with UV light through a photomask with an 8 mm *x* 8 mm opening (1.9 J/cm^2^). Exposing this control gel also through a mask ensured the same exposure conditions as the line samples. The completely covered gels were used as a positive control to confirm cell adhesion onto the RGD peptides and as a negative control sample for the cell orientation on the confined lines. Hyaluronic acid acrylamide gels exposed to UV light (1.9 J/cm^2^) through a mask and without the presence of RGD peptide solution was used as a negative control for cell adhesion.

### Cell culture

To obtain this data we used the mouse brain microvascular endothelial cell line bEnd.3 (CRL-2299, ATCC). The cell line was initially tested for mycoplasma contamination by the vendor and no contamination was detected. Furthermore, we regularly test the cultures ourselves and they have not been found contaminated.

The cells were cultured as suggested by the vendor, in Dulbecco’s Modified Eagle Medium Glutamax High Glucose (Gibco) supplemented with 10% fetal bovine serum (GE Healthcare Hyclone) and 1% Penicillin Streptomycin (Lonza) and maintained at 37 °C in 5% CO_2_. Cell media was replaced every two days and the cells were passaged, when reaching 80% confluency, using TrypLE™ Express enzyme (Gibco) for dissociation.

### Sample preparation

To obtain the data from the different line widths associated with this data descriptor, bEnd.3 cells (passage between 24 and 35) were seeded on the patterned and control hydrogels at a density of 20,000 cells/cm^2^. The cells were kept in the same medium as described above for the routine cultures. The samples were kept in culture at 37 °C in 5% CO_2_ for 24 h until they were analysed.

### Glucose level variations

To obtain data from simulated hypo- and hyperglycaemic conditions, the cells were seeded on the 10 µm wide lines, as described above. A diabetic effect is often simulated *in vitro* by elevating the glucose concentration of the cell culture medium to between 25 and 35 mM of glucose in the hyperglycemic case or lowered to 1 mM glucose in the hypoglycemic case. Normal glucose conditions in healthy homeostasis is simulated by adding 5.5 mM of glucose to the cell culture medium^5,13,14^. Thus, to simulate these three different conditions the cells were subjected to medium with either 1 mM (low), 5.5 mM (normal), or 25 mM (high) glucose levels.

To make the low glucose medium we used DMEM without glucose (Gibco) supplemented with 1 mM D-glucose (Sigma). For the normal glucose medium, we used DMEM low glucose (Gibco) containing 5.5 mM D-glucose. And for the high glucose medium we used DMEM Glutamax High Glucose (Gibco) medium containing 25 mM D-glucose. All three media were otherwise supplemented as described above.

The glucose concentrations of the medium were changed as the cells were seeded onto the line-patterned hydrogels. Cells were seeded at a density of 20,000 cells/cm^2^ and the samples were kept in culture at 37 °C in 5% CO_2_ for 24 h, before fixed an imaged.

### Fluorescent labelling

The samples were imaged after 24 h in culture. First, the samples were fixed using PBS with 2% paraformaldehyde. The samples were then blocked for 2 h with a blocking buffer containing 3% bovine serum albumin (BSA) and 0.2% IGEPAL (Sigma) in PBS. Next the samples were immunofluorescently labelled.

To fluorescently label zonula occludens 1 (ZO-1), a rabbit-anti mouse ZO-1 primary antibody (cat. no. 43–2300, Invitrogen) was dissolved to 1:100 in 50% blocking buffer (see above) diluted in PBS. 100 µL was added to each sample to completely cover the whole gel. The samples were then incubated overnight at 4 °C. Afterwards, the samples were washed three times with Triton X-100 (0.3%) in PBS. Next, goat anti-rabbit Alexa Fluor 568 (cat. no. A-11011, Invitrogen) was dissolved in 50% blocking buffer (see above). 100 µL was added to each sample to completely cover the whole gel and they were subsequently incubated for 2 h at room temperature (RT) in the dark. Afterwards, the samples were rinsed with Triton X-100 (0.3%) and washed two times with PBS.

F-actin was fluorescently labelled using SPY620-Actin (cat. no. SC402, Spirochrome). The label was diluted 1:1000 in PBS and 100 µL was added to each sample to completely cover the whole gel. Next the samples were incubated for 1 h at RT in the dark, followed by nuclei staining with Hoechst 33342, diluted 1:2000 in PBS, for 20 min at RT in the dark. Finally, the samples were washed two times with PBS. All steps were done sequentially.

### Confocal imaging

Cell images were acquired with a laser scanning confocal microscope (Leica SP8) equipped with a 25X/0.95 water immersion objective.

A 488 nm laser was used for excitation of the fluorescently conjugated RGD peptide lines a 488 nm, and the fluorescent emission was collected between 493 and 531 nm. To excite the Alexa Fluor 568 fluorescently labeled ZO-1, a 552 nm laser was used. The emission range was set to 578–603 nm. The SPY620-Actin-labeled F-actin was excited using a 638 laser and a set emission spectrum of 619–640 nm. Finally, the Hoechst 33342-labeled nuclei were visualized by using a 405 laser and emission was collected between 410 and 493 nm. The pinhole diameter was set to 1 airy unit throughout the data collection sessions.

A set of z-stacks were then collected from the samples with a fixed distance of 0.57 µm between the slices, as deemed to be optimal by the microscopy software. Care was taken to set the first and last focal planes out-of-focus on either side of the labelled cells. The stack resolution was set by choosing “optimal” from the microscopy software. The collected images have a frame size of x-2048 by y-2048 and a pixel size of 0.23 µm. The same image settings were applied to all collected images. The confocal stacks are found at 10.17605/OSF.IO/3G6S2^15^.

## Data Records

**Fig. 2:**
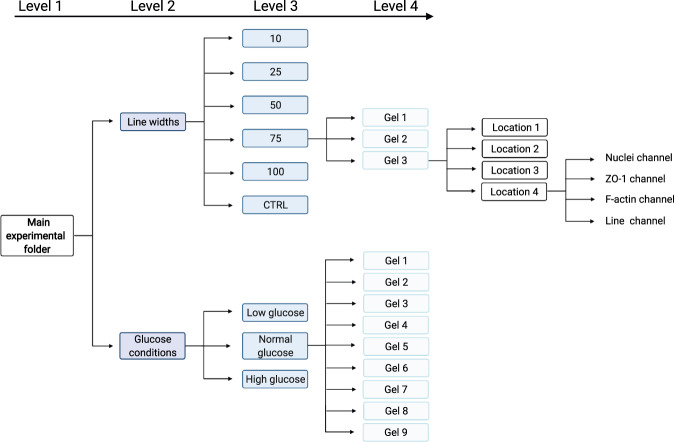
Overview of the folder structure in the data repository. An illustration of the folder structure from the main folder down to the individual stacks. Created with BioRender.com.

We have deposited the data records associated with this descriptor in the Open Science Framework repository (10.17605/OSF.IO/3G6S2)^15^. For an overview of the folder structure see Fig. 2. The data set size if 15 GB.

The image data is divided into two folders. The first folder contains the images of the cells on different line widths named “Line widths”, and another folder contains the images of the cells challenged under different glucose conditions named “Glucose conditions”.

Each main folder is then subdivided further, where the “Line width” folder subsequently contains one folder for each line width and the control condition where the whole surface is covered with the adhesion peptide, named “10”, “25”, “50”, “75”, “100”, and “ctrl”. The “glucose conditions” folder is equally subdivided into three folders representing the different glucose conditions named “Low glucose”, “Normal glucose”, and “High glucose”. Every level 3 folder then contains one folder of each gel run for this condition. For each gel four different parts of the gel have been imaged in four different colors (except for the control samples where no lines are imaged as these cells are not cultured on lines). Consequently, each gel folder contains confocal stacks of 4 locations x 4 fluorescent channels. Note that the line images, corresponding to the green channel, has only been collected as a single image and not a stack. This is because the lines don’t contain any interesting spatial information but rather works as a reference for the cell or cellular structure orientation of the other channels.

Finally, the main folder also contains an excel sheet detailing the experimental conditions for each image stack.

## Technical Validation

To validate the quality of our images we produced maximum intensity projections of each stack and run them through the CellProfiler^TM^ (version 4.2.1)^16^ module *MeasureImageQuality*. The maximum intensity projections were produced using Fiji^17^.

**Fig. 3:**
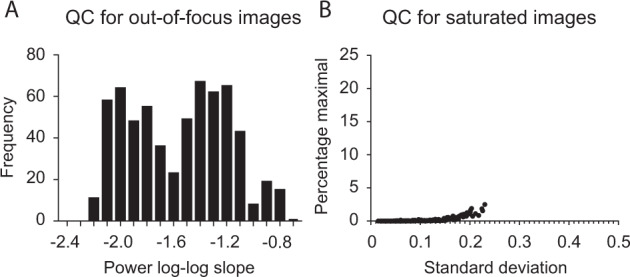
Image quality control metrics. (**A**) Quality control for out-of-focus images showing the power log-log slope. A cut off value for out of focus images is below −2.3. (**B**) Quality control for saturated images showing the percentage of pixels at maximum intensity as a function of the standard deviation of the pixel intensities. No images stand out as having a high percentage of pixels at maximum intensity or a high standard deviation.

To determine if we had any out-of-focus images we used the power log-log slope (PLLS), that shows the slope of the power spectrum density of the pixel intensities on the log-log scale. We specifically chose this metric as it is not as sensitive to the cell count of the image, as we only have cells in spatially confined areas of our images, and there are more cells in the images with wider lines. The resulting PLLS values are always negative and decrease as the blur increases. Outliers that are less than about −2.3 are defined as blurry images^18^. Among the images included in this dataset none of the images fell below this cut off (Fig. 3A).

Next, we evaluated our dataset for the presence of saturated artifacts *e.g*., debris or other contaminations but also inappropriate exposure or gain settings, producing inappropriately bright regions in the images. To assess this metric, we utilized the percent maximal which shows the percentage of pixels that are at maximum intensity values. In addition, the standard deviation of the pixel intensity is also a useful measure to detect images where brighter artifacts may appear but are not bright enough to cause substantial pixel saturation^19^. Here, we have plotted the percentage of pixels at maximum intensity as a function of the standard deviation of the pixel intensity. As can be seen in Fig. 3B none of the images in this dataset stand out as having a high percentage of pixels at maximum intensity or a high standard deviation of the pixel intensity.

## Usage Notes

The data available in this descriptor can for example be extracted and analyzed by the mean of using free software such as Cell Profiler^TM^, Cell Profiler Analyst, or ImageJ. More advanced image analysis can also be performed utilizing for example MATLAB, Python or OpenCV.

## Data Availability

No custom code was used to generate or process the data described in this data descriptor.
